# Cardiac Involvement in COVID-19 Patients: A Contemporary Review

**DOI:** 10.3390/idr13020048

**Published:** 2021-06-01

**Authors:** Domenico Maria Carretta, Aline Maria Silva, Donato D’Agostino, Skender Topi, Roberto Lovero, Ioannis Alexandros Charitos, Angelika Elzbieta Wegierska, Monica Montagnani, Luigi Santacroce

**Affiliations:** 1 AOU Policlinico Consorziale di Bari-Ospedale Giovanni XXIII, Coronary Unit and Electrophysiology/Pacing Unit, Cardio-Thoracic Department, Policlinico University Hospital of Bari, 70124 Bari, Italy; carrettacardiologia@gmail.com; 2 AOU Policlinico Consorziale di Bari-Ospedale Giovanni XXIII, Cardiac Surgery, Policlinico University Hospital of Bari, 70124 Bari, Italy; dr.alinemariasilva@gmail.com (A.M.S.); prof_d.dagostino@libero.it (D.D.); 3 Department of Clinical Disciplines, School of Technical Medical Sciences, University of Elbasan “A. Xhuvani”, 3001 Elbasan, Albania; skender.topi@uniel.edu.al; 4 AOU Policlinico Consorziale di Bari-Ospedale Giovanni XXIII, Clinical Pathology Unit, Policlinico University Hospital of Bari, 70124 Bari, Italy; robertolovero69@gmail.com; 5 Emergency/Urgent Department, National Poisoning Center, Riuniti University Hospital of Foggia, 71122 Foggia, Italy; 6 Department of Interdisciplinary Medicine, Microbiology and Virology Unit, University of Bari “Aldo Moro”, Piazza G. Cesare, 11, 70124 Bari, Italy; angelika.wegierska@yahoo.it; 7 Department of Biomedical Sciences and Human Oncology—Section of Pharmacology, School of Medicine, University of Bari “Aldo Moro”, Policlinico University Hospital of Bari, p.zza G. Cesare 11, 70124 Bari, Italy; monica.montagnani@uniba.it

**Keywords:** SARS-CoV-2, viral myocarditis, cardiomyopathy, endomyocardial biopsy, cardiac magnetic resonance, cardiovascular therapy

## Abstract

*Background*: The widely variable clinical manifestations of SARS-CoV2 disease (COVID-19) range from asymptomatic infections to multiple organ failure and death. Among the organs affected is the heart. This does not only affect people who already have previous cardiovascular problems, but also healthy people. This is a reason not to overlook any symptoms or to perform targeted examinations, even if apparently unrelated to the heart, for quick recognition and timely therapy. *Aim of the study*: This review recapitulates the current state of knowledge on the potential mechanisms and manifestation of myocarditis in patients with COVID-19 infection. *Methods*: A web-based search of published data was performed for all relevant studies on patients diagnosed with a COVID-19-induced acute myocarditis, and a total of 50 reports were included. The analysis of the studies evaluated highlights a male predominance, with the average age of patients being 55 years. The most common presenting symptoms included fever, shortness of breath, cough, and chest pain. Among ECG changes, non-specific ST-segment and T-wave amplitude alterations and ventricular tachycardia episodes were reported. Finally, we wanted to use a general evaluation without distinguishing between various countries, taking into consideration only the peer or reviewer, regardless of the declared value of the journals that have been published. *Results* and critical findings: The most common presenting symptoms included fever, shortness of breath, cough, and chest pain. Among ECG changes, non-specific ST-segment and T-wave amplitude alterations and ventricular tachycardia episodes were reported. In most patients, elevated levels of cardiac and inflammatory biomarkers were measured. Left ventricular dysfunction and hypokinesis were commonly exhibited symptoms. Cardiac Magnetic Resonance Imaging (CMRI) confirmed the diagnosis of myocarditis with features of cardiac edema and cardiac injury. Nine patients underwent histopathological examination. Treatment with corticosteroids and immunoglobulins was the most applied strategy following the administration of antivirals. *Discussion*: Despite the exponentially growing knowledge on the management of COVID-19 infection, current available data on SARS-CoV2-correlated myocarditis are still limited, and several difficulties may be encountered in the differential diagnosis of acute myocarditis in the context of COVID-19 disease. *Conclusions*: While diagnostic criteria and evaluation strategies for myocarditis are well described, no guidelines for the diagnosis and treatment of myocarditis in COVID-19 patients have yet been established. Therefore, further research is needed to advance the understanding of this disease process and define the most appropriate strategic approach in these patients.

## 1. Introduction

Severe acute respiratory syndrome coronavirus 2 (SARS-CoV-2) is a new variant form of coronavirus identified as the pathogen responsible for coronavirus disease 2019 (COVID-19). SARS-CoV-2 is like the severe acute respiratory syndrome coronavirus (SARS-CoV) and the Middle East respiratory syndrome coronavirus (MERS-CoV), and is believed to be the result of zoonotic transmission to humans from bats [1,2,3].

In December 2019, a COVID-19 outbreak was first reported in Wuhan, China, and in March 2020, the World Health Organization (WHO) declared a COVID-19 global pandemic after confirming cases on five continents [3,4]. 

Millions of COVID-19 cases have been reported so far, and efforts are underway to better define the epidemiology of the disease. Clinical manifestations of COVID-19 are widely variable and range from asymptomatic infection to multi-organ failure and death [4,5,6]. 

Although pulmonary involvement is the most common and most virulent clinical manifestation, cardiovascular symptoms have been found in 30% of hospitalized patients, sometimes associated with worse outcomes [6,7,8]. The understanding of the pathophysiology for the occurrence of myocarditis in COVID-19 is currently limited to clinical case reports. This review recapitulates the current knowledge on the potential mechanisms and manifestations of heart damage, and specifically of myocarditis, in patients with COVID-19 infection, in the attempt to clarify whether they were caused by direct viral damage or were secondary to inadequate host immune response. Here, clinical reports of myocarditis in COVID-19 patients are summarized and described to help shed light on this field, and increase our understanding of the clinical features, diagnostic process, and outcomes of this rare but often serious disease complication [9,10,11,12,13,14,15,16].

## 2. Methods

A web-based search of the published medical literature was performed for all relevant studies on patients diagnosed with COVID-19-induced acute myocarditis using the LitCovid, PubMed, Cochrane Library, Embase databases, Google Scholar, Elsevier Connect, and Web of Science, using the following keywords: “COVID-19”, “coronavirus”, and “SARS-CoV-2”, in combination with the term “myocarditis”. The identified reports were independently screened by two investigators (AMS and IAC). Only papers written in English were included. Each paper was retrieved, and its references were reviewed to identify additional studies. For each case, age, gender, clinical presentation, laboratory results, electrocardiogram, echocardiography, cardiac imaging, and endomyocardial biopsy data were considered. Only articles reporting myocarditis or myocardial infection in association with COVID-19 were included. Articles were initially selected based on whether the titles or abstracts met the inclusion criteria. Full-text articles that did not meet the appropriate inclusion criteria were excluded. Data extracted from articles include first author name, study design, and study sample.

## 3. Results and Critical Findings

For this review, 1296 articles were initially found by searching the indicated databases with the above-mentioned key words, and 864 studies underwent further evaluation after the removal of duplicates. Among the 65 articles meeting the inclusion criteria were 15 reviews, 36 case reports, 3 case series, 2 cohort studies, 5 retrospective studies, 2 meta-analyses, 1 single-center study, and 1 prospective study. All articles were published in 2020. Studies were carried out in Asia, Europe, and America. Table 1 and Table 2 below summarize the results from the published studies.

In March 2020, two published studies gave the first glimpse of how prevalent heart problems were among patients with COVID-19 disease. The larger of these two studies included 416 hospitalized patients. Among them, a 20% incidence of heart damage was observed; the mortality reported was 51.2% in patients with cardiac damage, and 4.5% in those without it [63].

Similarly, in another study, heart damage occurred in 27.8% of 187 COVID-19 patients, and the presence of heart damage was associated with fatal outcomes, that is, with a significantly higher mortality (59.6%) compared to that observed in patients without heart damage (8.9%) [55]. In an observational cohort study of German patients who recently recovered from COVID-19 disease and underwent CMRI (with a mean duration of 71 days after COVID-19 diagnosis), 78% of patients displayed features of heart disease, and 60% showed signs of ongoing inflammation, regardless of the severity and general course of the disease [60]. This suggests that a potential preexisting SARS-CoV-2 infection should be considered in patients with unexplained acute heart failure or new-onset arrhythmias detected by cardiac electrophysiology procedures. Endomyocardial biopsy (EMB)-based identification of the causes underlying myocardial damage may help explain the diverse evolution of complicated SARS-CoV-2 infection and support the design of future specific and personalized treatment strategies [65,66,67].

The analysis of the available studies highlights a male predominance (69%), with the average age of the cases described being 55 years, and with differences between case reports and retrospective studies. COVID-19 patients with myocarditis were 50 ± 16 years old, and males and females were equally affected in the case report studies [17,18,19,20,21,22,23,24,25,26,27,28,29,30,31,32,33,34,35,36,37,38,39,40,41,42,43,44,45,46,47]. In contrast, retrospective studies described the typical myocarditis patient as male and older than 55 years. One third of all cases were under the age of 40. Three cases were under the age of 18, with two cases of children under 13 years old. Most patients do not have a previously identified co-morbid condition; hypertension is the most frequently observed risk factor. In general, for these patients, the earliest and most common predominant symptom was dyspnea along with fever, followed by cough and chest pain. Other reported symptoms include fatigue, nausea and vomiting, diarrhea, myalgia, weakness, and headache [52,53,54,55,62].

### 3.1. ECG Results

Almost all patients showed ECG features consistent with myocarditis, such as ST-segment changes including elevation and depression, T-wave inversions, as well as ventricular tachycardia, nonspecific intraventricular conduction delay, and multiple premature ventricular complexes [52,55,58,60]. Levels of troponin (troponin I or troponin T) were elevated (with remarkably high values in some patients) almost constantly [21,25,27,29,30,34,37,38,53,57,60]. N-terminal pro B natriuretic peptide (NT-pro-BNP) was elevated in all cases in which values were measured. Inflammatory markers including white blood cell counts, interleukin-6 levels, and C-reactive protein levels were elevated in all patients (see Table 1 and Table 2).

### 3.2. Results of Cardiac Ultrasound and Advanced Imaging

Echocardiography was performed in most cases and revealed a decrease in left ventricular ejection fraction (LVEF). Patients with severely impaired left ventricular function developed cardiogenic shock, and thus, had a worse prognosis. Cardiomegaly or increased wall thickness and pericardial effusion have also been observed, and the presence of cardiac diffuse hypokinesia is registered in 30% of cases (see Table 1). Cardiac magnetic resonance (CMR) was not performed in all cases, due to the prolonged acquisition time and high contagiousness of COVID-19 infection, but in most cases, myocarditis was diagnosed on the basis of Lake Louise criteria, which included signs of myocardial edema on T2-weighted imaging and myocardial damage on T1-weighted imaging with gadolinium enhancement [19,20,21,22,24,25,28,29,32,33,35,37,38,41,42,44,45,46,60,61]. Bilateral infiltrates and ground glass opacities were observed in all patients undergoing computed tomography (CT) of the chest [52].

### 3.3. Histopathological Tests Results

Few cases of myocarditis diagnosed by histopathological or imaging tests that met the Lake Louise consensus criteria were confirmed in COVID-19 patients [46]. For several others, they remain possible cases, in which the authors reported a diagnosis of myocarditis, with or without support for serological, electrocardiographic, and echocardiographic characteristics. Endomyocardial biopsy (EMB), although considered a gold standard for the diagnostic confirmation of myocarditis, has not been performed in all patients with suspected COVID-19 myocarditis for various reasons, such as the associated coagulopathy and hemodynamic instability. Despite this, in the few cases where it was performed, it showed virus-negative lymphocytic myocarditis, except in seven patients, one from autopsy after sudden death and six from endomyocardial biopsy for clinical reasons. In a patient with hypokinesia and diffuse myocardial edema to the basal and middle segments of the left ventricle on CMR, the EMB showed diffuse inflammatory T-lymphocytic infiltrates. There was massive interstitial edema and limited foci of necrosis, meeting the Dallas criteria. However, the SARS-CoV-2 genome was not identified within the myocardium, suggesting a diagnosis of virus-negative acute lymphocytic myocarditis. A second case was a previously healthy 17-year-old African American male who died on arrival at the hospital after complaining of severe headaches, dizziness, nausea, and vomiting for two days. Pathological examination of the heart revealed diffuse inflammatory infiltrates composed of prominent lymphocytes, macrophages, and eosinophils, mainly in the interstitial, and associated with multiple foci of myocyte necrosis. This was identified in both ventricles, and eosinophilic myocarditis was diagnosed. Subsequently, in a study by F. Escher et al., the presence of the SARS-CoV-2 genome was reported in five patients with myocarditis undergoing EMB. From several studies, it is not clear how the viral load or the patient’s immunopathology relate to different cardiac pathological findings. Histological and ultrastructural findings in a serious case study reported, from autopsies of mostly elderly fatal cases with significant comorbidities, viral particles (case-to-case variability) found in the heart, large intestine, liver, spleen, kidney, trachea, subcarinal lymph nodes, and cardiac tissue of a patient with lymphocytic myocarditis. The presence of the virus has not always been associated with increased infiltration of mononuclear cells into the myocardium [23,24,30,41,43,54,56,59].

### 3.4. Therapies and Advanced Treatment

Medical management includes therapies targeting COVID-19, therapies for the treatment of myocarditis, and therapies to control the cytokine storm. Corticosteroids were the most used drugs in the treatment attempt, followed by the antiviral drugs lopinavir and ritonavir. The use of hydroxychloroquine, immunoglobulins, piperacillin-tazobactam, and azithromycin was also observed. The use of inotropes and/or vasopressors was reported in 50% of studies. Additional therapies targeting the cytokine storm, such as tocilizumab and interferon, were also employed. Some patients with COVID-19-induced acute fulminant myocarditis have improved after intravenous glucocorticoid and immunoglobulin therapy. This last finding advocates for the cytokine storm being a result of an inadequate host immune response, which could be the main pathophysiological explanation of COVID-19-induced acute fulminant myocarditis. Mechanical support via extracorporeal membrane oxygenation (ECMO) was also used. There is no common consensus for prescribing standard treatment for heart failure in addition to antiviral therapy to hospitalized patients with COVID-19-induced acute myocarditis. In several cases, this last condition was treated with diuretics, angiotensin converting enzyme (ACEi) inhibitors, and beta blockers (see Table 1 and Table 2).

## 4. Discussion

### 4.1. Biomolecular Hypotheses about COVID-19-Induced Myocardial Injury

**Figure 1:**
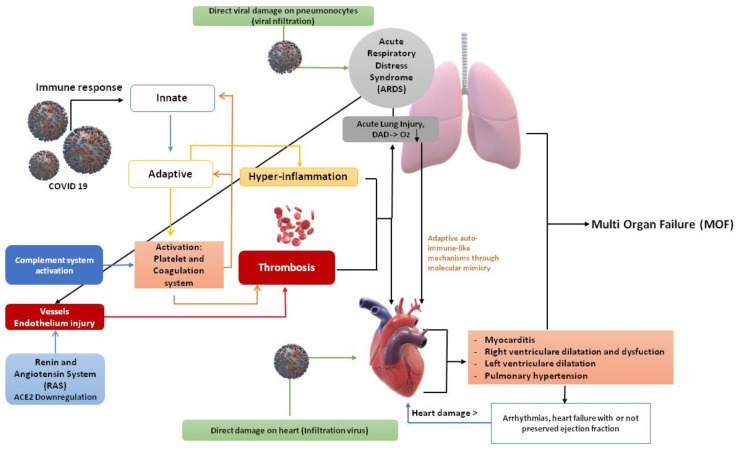
The myocardial damage hypothesis. Although the predominant injury can be ascribed to the wide-spreading inflammation in the context of COVID-19 infection, a direct heart damage by the virus itself is not excluded. According to this hypothesis, SARS-CoV-2 can directly and indirectly affect both pneumocytes and myocardiocytes, concomitantly leading to a severe injury of lung and heart tissues. The initial activation of the innate immune response, and the subsequent recruitment of the adaptive immune system (CD8 and B antiviral cells, antibodies) with the contribution of the complement system (such as C3a, C5a, membrane complex) may explain the hyper-inflammation (by cytokines/chemokines storm) and its deleterious consequences for lung and heart tissues. In addition, downregulation of ACE2 receptors increases ACE/AT1, which leads in turn to vasoconstriction and endothelial damage in both lung and heart. Activation of the coagulation system and dysregulation of ACE2 will also amplify the risk of clot formation, with enhanced thrombosis due to a hypercoagulable state. This condition increases the possibility of diffuse alveolar damage (DAD), aggravates the severe hypoxemia, and triggers an acute respiratory distress syndrome (ARDS) on one side, but may also aggravate an underlying heart dysfunction and induce myocardial ischemia and heart damage. Severe hypoxemia may progressively worsen the harmed myocardium and result in acute myocarditis, dilatation of the left and/or right ventricle (in this case leading to pulmonary hypertension), or impaired electrophysiological and structural heart remodeling. The co-existence of myocardial inflammation and interstitial edema with cardiac fibrosis, together with an abnormal regulation of the intracellular Ca^++^ and K^+^ concentrations can facilitate arrhythmias (especially tachyarrhythmias) and subsequently heart failure, further damaging the heart. In the worst scenario, the overall systemic damage may lead to a multi-organ failure (MOF) [1,9,78,80,81,82,83] Credits: Original figure by I.A. Charitos).

SARS-CoV-2 is a beta coronavirus whose genome consists of single-stranded ribonucleic acid (RNA) with positive polarity that belongs to the Coronaviridae family. The virus can invade the human host cell by binding with high affinity to the angiotensin converting enzyme 2 (ACE 2) receptor. ACE-2 is widely expressed throughout the body, including in the lungs and heart. After penetration, viral RNA enters the cell nucleus for replication and apoptosis [1,68,69]. The human immune response to the virus is individual, which explains its variable clinical presentation. The virus mainly infects the lung epithelium with respiratory signs and symptoms, but there have been cases with COVID-19-induced acute myocarditis. The mechanism of heart damage remains poorly understood. Currently, several mechanisms have been proposed to explain the underlying pathophysiology of COVID-19-related acute myocarditis. Among them, the main ones are the following (Figure 1):

(a) Myocardial damage due to the *direct viral action on the tissue*, or to extrapulmonary migration of infected alveolar macrophages. Thus, a direct myocardial damage through binding to ACE2 receptors because of viral action on the tissue or due to extra pulmonary migration of infected alveolar macrophages. SARS-CoV-2 uses spike protein to bind to ACE2 receptors on the myocardial cell membrane. In theory, following SARS-CoV-2’s engagement to the ACE2 receptors on the myocardium and the subsequent down-regulation of the receptors themselves, the accumulation of angiotensin II may enhance myocardial remodeling by diverting all its activity to type 1 angiotensin II receptors (AT1R) of the heart [1,70,71]. Interestingly, a polybasic furin site that structurally differentiates SARS-CoV-2 from SARS, when cleaved, expands the types of cells the virus can infect. The virus, by binding to ACE2 receptors throughout the body, facilitates cell entry through its spike protein, together with the cooperation of the serine protease transmembrane protease serine 2, heparan sulfate, and other proteases. The heart is one of the many organs with high ACE2 expression [72,73,74,75]. Tropism to other organs besides the lungs was studied from autopsy samples: SARS-CoV-2 genomic RNA was highest in the lungs, but the heart, kidneys, and liver also showed substantial amounts, and copies of the virus were detected in the heart of 16 of the 22 patients who died. In an autopsy series of 39 patients who died from COVID-19, the virus was undetectable in the myocardium in 38% of patients, while 31% had a high viral load greater than 1000 copies in the heart [76,77].
(b) Via *cell-mediated cytotoxicity* in which CD8 T lymphocytes migrate to cardiomyocytes and cause inflammation of the myocardium. Cytokine release syndrome, proposed as the main mechanism underlying COVID-19-induced acute fulminant myocarditis, is thus unleashed [1,9,10,11]. This triggers the pro-inflammatory cytokine release syndrome, a severe systemic inflammatory response resulting in hypoxia and apoptosis of the cardiomyocytes. In fact, the released cytokines increase the activation of T lymphocytes, which release further cytokines with consequent positive feedback of immune activation and myocardial damage [9].
(c) The mechanism of *hyperactivation of the autoimmune system* with possible interferon-mediated hyperactivation of the innate and adaptive immune system has also been proposed [78]. Myocarditis can cause the onset of arrhythmias, during the acute phase, due to a direct cytopathic effect, which causes electrical imbalance and ischemia due to microvascular and gap-junctions dysfunction due to reduced myocardial expression of connexins [79]. Thus, the main biomolecular mechanism responsible for heart damage, and in turn heart failure, is an abnormal state of systemic inflammation with low oxygen levels and thrombosis. In addition, the presence of the SARS-CoV-2 genome was detected in the myocardium [54].

Although rare, serious systemic infections such as myocarditis have been reported in connection with COVID-19, myocarditis, an inflammatory condition affecting the myocardium, arises from a broad spectrum of both infectious and non-infectious causes. Many different viruses have been implicated, including the Middle East Respiratory Syndrome (MERS) coronavirus, which closely resembles SARS-CoV-2 [84,85,86,87]. Myocarditis is suspected based on elevated troponins in the patient’s blood, cardiac arrhythmias or diffuse ST-segment elevation on electrocardiogram (ECG), and impaired contractility of the left ventricular wall (regional or global hypokinesia) on echocardiography. Clinical manifestations of myocarditis include subclinical, subacute, acute, and fulminant forms, and sudden onset myocarditis is known to be associated with significant severity [88,89,90]. After reviewing the literature and collecting reports related to COVID-19-associated viral myocarditis, it is interesting to note that the diagnosis of viral myocarditis was made on clinical and imaging grounds in several cases of COVID-19. If the predominant injury is caused by widespread inflammation in the context of COVID-19 infection, direct damage to the heart by the virus itself is not excluded.

Some studies have documented the presence of interstitial inflammation of the myocardium and edema on CMRI, while others have reported interstitial inflammatory infiltrates based on EMCB. However, the SARS CoV-2 genome was isolated in very few cases only under EMCB. Therefore, although various non-invasive investigation methods such as echocardiography and nuclear magnetic resonance allow evaluation of the structural and functional alterations of the heart, providing valuable information useful in the diagnosis and prognostic stratification of this disease, the diagnosis of certainty and the complete characterization of the pathological substrate of myocarditis can only be obtained by means of EMCB, an invasive investigation that is critical for planning a specific therapy (see Table 1 and Table 2). Nevertheless, the feasibility of performing EMCB in all suspected cases is questionable, especially considering the risk of contagious spread, the skill required, and the false negative rate. Furthermore, during this type of pandemic, it is not feasible to perform an EMCB only to confirm or rule out acute viral myocarditis in patients with signs and symptoms of acute heart failure, even based on suggestive features by CMRI, and without other plausible causes. This limitation has been partially overcome, where possible, with autopsies in patients with suspected Covid-19 myocarditis to detect the presence of viral genomes. To date, although clinical data on myocarditis caused by SARS-CoV-2 are limited, patient characteristics, clinical course, degree of severity, imaging and electrocardiographic hallmarks, and management and outcomes of myocarditis are particularly relevant, and should be considered by physicians when facing patients with potential COVID-19 myocarditis. However, the potential pathogenetic significance of this finding is still unclear [54,70,91,92,93,94,95].

### 4.2. Cardiovascular Signs and Symptoms during the Infection

**Figure 2:**
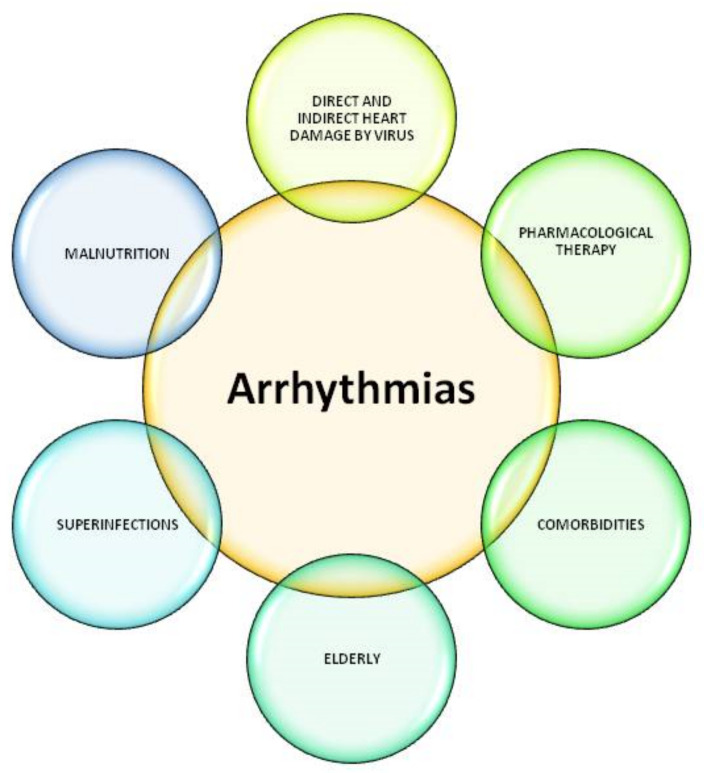
The main modifiable and non-modifiable risk factors that may predispose patients to arrhythmias. Several arrhythmias may arise in patients with myocarditis during SARS CoV-2 infection, especially in the critical care setting. Among them, both bradyarrhythmias (such as atrioventricular nodal block, sinus node dysfunction, and sinus bradycardia) and tachyarrhythmias (such as ventricular tachycardia, atrial fibrillation, atrial flutter, atrial tachycardia, sinus tachycardia) may occur. Inflammatory cytokines acting on both cardiomyocytes (especially interleukin 6) and autonomic nervous system (ANS) can promote QTc prolongation. The administration of antivirals (such as protease inhibitors lopinavir/ritonavir), antibiotics (such as macrolides, fluoroquinolones, and others), and the antimalarials (such as hydroxychloroquine) can trigger similar consequences. Finally, cardiometabolic risk factors (such as obesity, diabetes), recent or existing cardiac diseases (previous myocardial injury, atrial fibrillation, hypertensive heart disease, and others), and chronic pneumopathies (chronic obstructive pulmonary disease (COPD) and others) are examples of the comorbidities that increase the risk of heart damage and therefore favor the onset of malignant arrhythmias.

A variety of cardiac disturbances have been observed in the COVID-19 pandemic, including acute myocardial infarction, acute heart failure, cardiogenic shock, myocarditis, and malignant arrhythmias. It must be emphasized that the viral infection can not only deregulate a pre-existing heart disease, but also exacerbate mild asymptomatic pre-existing heart failure with long-term complications. The increased risk of complications in patients with heart failure, various arrhythmias, post-infarction, and similar vulnerable conditions leads to a higher risk for suspected myocardial damage (Figure 2) [81,82,83].

Patients with underlying cardiovascular disease (CVD) have an increased risk of developing heart damage [6,74,75]. In a retrospective single-center study that included 187 patients, the mortality rate was higher in patients with underlying CVD than in patients without CVD (54.5% vs. 13.2%) [55]. In order of prevalence, the clinical manifestations of severe acute respiratory syndrome due to COVID-19 are fever, cough, and fatigue [6]. Typically, myocarditis arises with fever, dyspnea, and chest pain, all symptoms which hinder the recognition and clinical diagnosis of myocarditis during the COVID-19 pandemic [7,96]. Indeed, our systematic revision of selected studies confirms that in patients with COVID-19, myocarditis commonly starts with dyspnea, followed by cough, fever, and chest pain. Other reported symptoms include fatigue, nausea and vomiting, diarrhea, myalgia, weakness, and headache.

### 4.3. Main Laboratory Markers of Cardiovascular Severe Prognosis

Serum biomarkers are conventionally used to confirm suspected acute myocarditis. While troponin was elevated in some patients with acute myocarditis, elevated serum cardiac troponins were almost always seen in fulminant myocarditis. Elevated troponin levels have been described in many COVID-19 patients, with differences observed between deceased and surviving patients. The absence of an increase in serum cardiac troponins, however, does not rule out myocarditis [6,7,78,83]. The retrospective study of a single Wuhan center that included 187 patients showed that 37.5% of deceased patients had heart damage with elevated troponin, and mortality was 69.4% in those with a history of CVD [55]. In most studies, cardiac damage was associated with elevated troponin and pro-BNP amounts. Levels of these markers were higher in critically ill patients admitted to intensive care. Levels of creatine kinase myocardial band (CKMB) and BNP were often elevated in myocarditis and can provide information on prognosis. CRP was commonly elevated, although normal levels did not exclude myocarditis. However, CRP has also been shown to be above the normal range in most COVID-19 patients, and increases in troponins, CK-MB, and BNP [7,78,83]. It is important to underline an aspect that represents a limitation in the differential diagnosis of COVID-19 myocarditis. Troponin has been shown as an independent predictor of mortality in hospitalized patients with COVID-19. In COVID-19 patients who have elevated troponin levels, it may be difficult to reach a definitive diagnosis of acute coronary syndrome or acute myocarditis because there are similarities between them regarding elevated troponin levels and changes in ECG. It is reasonable not to perform MRI in most patients due to the contiguity of COVID-19. On the other hand, the underuse of the modalities in the differential diagnosis reveals a gap in the identification of the definitive etiology of myocardial damage (see Table 1 and Table 2). Therefore, we may underestimate the prevalence and importance of COVID-19-related myocarditis. Additionally, in severe patients, both acute coronary syndrome (ACS) and acute myocarditis can occur together due to the procoagulant and inflammatory nature of the COVID-19 infection [1,6,7,78,97].

Finally, some studies report that the laboratory results showed differences in white blood cell counts and specifically in lymphocytes count. Indeed, in a retrospective multicenter study for clinical predictors of mortality in patients who had died (N = 68), white blood cells (×109/l) were 10.62 with lymphocyte counts (×109/l) 0.60 (0.32); instead, in the discharged patients (N = 82) white blood cells were 6.76 (3.49) and lymphocytes 1.42 (2.14), with a *p*-value < 0.001 for both two groups. In another retrospective single-center case study with 187 patients, it was found that Troponin T levels and NT-proBNP levels were significantly increased in deceased patients. These patients had higher white blood cell and neutrophil counts and lower lymphocyte counts than those with normal Troponin T levels [54,55,62]. Therefore, there may be a correlation between lymphopenia and myocarditis severity, but this needs further investigation to confirm it.

### 4.4. Instrumental Diagnostics

ECGs of most patients with myocarditis have demonstrated nonspecific features including sinus tachycardia, ST-segment, and T-wave abnormalities, and occasional atrioventricular or branch blocks. Although ST-segment elevation in segmentally contiguous leads or in a non-vascular distribution are common electrocardiographic signs of myocarditis, they can often be mistaken for coronary occlusion [7,74,83,96,98]. Based on studies reported here, ECG changes were non-specific and highly variable. This is consistent with the non-specific ECG findings in myocarditis reported in the literature. Serial ECGs can be a tool to provide a relatively quick, affordable, and non-invasive means of delineating and intervening in the early stages of the disease process (see Table 1 and Table 2).

Echocardiography is important in the assessment of structural and functional changes secondary to myocarditis. Although there are no specific echocardiographic features of myocarditis, it might nevertheless allow the physician to rule out other causes of heart failure, including pericardial effusion, and intracavitary thrombi [98,99,100,101].

Cardiac MRI is the non-invasive gold standard test for myocarditis. It was performed in a few of the reported cases, all of which showed an increase in signal intensity after gadolinium. Some showed evidence of myocarditis as fulfilled by the Lake Louis criteria for the diagnosis of myocarditis, such as edema and myocardial damage [60,101].

### 4.5. Histological Findings of Myocarditis

In histological diagnosis of myocarditis, echocardiographic patterns of dilated, hypertrophic, restrictive, and ischemic cardiomyopathies have been described, especially left ventricular dilation, and increase in volume. The reduction in LVEF is an independent predictor of a fulminant disease course [7,102]. Some reviews report that most patients with COVID-19 myocarditis had reduced LVEF, cardiomegaly, and pleural effusion. Dilated ventricles are typical of acute myocarditis, although the fulminant form can lead to an increase in the thickness of the left ventricular wall because of active inflammation [102]. Endomyocardial biopsy (EMB), although considered a gold standard for the diagnostic confirmation of myocarditis, has not been performed in all patients with suspected COVID-19 myocarditis for various reasons, but remains the key test to detect the presence of the SARS-CoV-2 genome (see Table 1 and Table 2).

### 4.6. Management and Treatment Strategies

Among COVID-19 patients, there is limited evidence of the effectiveness of current management and treatment strategies [6]. Several treatments have been used in COVID-19 myocarditis based on our understanding of the pathogenesis and previous experience in the treatment of viral and fulminant myocarditis. Since cytokine release syndrome is a probable mechanism of injury in COVID-19 myocarditis, glucocorticoids have been used despite the lack of proven clinical efficacy [103,104]. Data on the use of glucocorticoids in COVID-19 infection appear controversial. In a Wuhan study involving 84 patients with ARDS secondary to COVID-19, administration of corticosteroids reduced the risk of mortality. Additionally, in a recent press release from RECOVERY (Randomized Evaluation of COVid-19 thERapY), a large clinical trial in COVID-19 patients, dexamethasone was shown to reduce mortality in one-third of ventilated patients. In a recent review of COVID-19-associated myocarditis, a study was reported in which five out of seven patients who had been treated with corticosteroids recovered; one died, and the other case reported no results. We cannot confirm if it’s due to a true treatment effect or just chance, but based on recent data, the steroid may be associated with favorable outcomes in critically ill COVID-19 patients [105,106,107]. Some patients with COVID-19-induced acute fulminant myocarditis have shown improvement using intravenous (IV) administration of glucocorticoid and immunoglobulin therapy. Combined steroid treatment with immunomodulatory therapy and antibiotics was used in a minority of studies. IV immunoglobulin (IG) administration has been used with inconsistent outcomes. There is strong evidence for the efficacy of IV-IG in the treatment of acute myocarditis. A meta-analysis published in 2019 comparing IV-IG with corticosteroids for acute myocarditis showed that IV-IG therapy improved mortality and recovery of left ventricular function. There are only a few blinded randomized prospective studies that have specifically targeted viral infections or inflammatory cascades associated with myocarditis. Currently, the evidence does not support the routine use of IV corticosteroids or IV-IGs alone [108,109,110].

Tocilizumab is an IL-6 receptor antagonist used most in the treatment of rheumatoid arthritis. It is also approved for use in cytokine release syndrome based on multiple studies that have shown its effectiveness. In several cases on different studies examined it gave erratic results.

Other possible treatments are plasma exchange therapy, immunosuppression with IV-IG and cytokine inhibitors, and antiviral agents such as remdesivir. In the early results of the studies, remdesivir was found to be superior to placebo in shortening healing time in adults hospitalized with COVID-19. Until prospective studies and trials establish guidelines for the management of COVID-19 myocarditis, treatment must be tailored to the presentation of individual cases [110,111,112,113].

The guidelines of the European Society of Cardiology Working Group on Myocardial and Pericardial Diseases recommend optimal management of heart failure and arrhythmias caused by myocarditis, including the use of diuretics, ACE inhibitors, beta-adrenergic blockade, antiarrhythmics, and temporary stimulation [114].

The use of extracorporeal membrane oxygenation has been shown to provide favorable outcomes for patients with myocarditis and sepsis, as well as fulminant myocarditis [115,116,117].

Immunosuppression with the use of steroids, azathioprine, and cyclosporine is often attempted, but guidelines recommend starting only after active polymerase chain reaction infections have been established [6,7,118].

At present, given the lack of results from randomized control studies, the effectiveness of the various treatment strategies currently attempted in cases of COVID-19 myocarditis remains uncertain.

### 4.7. Knowledge Gaps and Future Directions

Due to the heterogeneous and not always consistent results from clinical studies, the current guideline recommendations on the most appropriate clinical management in patients diagnosed with myocarditis are extremely general. The intriguing question on why some individuals may possess a higher predisposition to develop a heart involvement after SARS-CoV-2 infection is still unanswered. A few months after the pandemic began, a cardiac involvement would be predicted mainly in patients with severe COVID-19 infection. However, present data suggest that this condition is more common than expected, although the exact incidence is unknown. It is therefore crucial to determine the most important factors involved in the pathogenesis of myocarditis and to clarify whether it may develop as an individual inflammatory response, an autoimmune phenomenon, or for some other reasons still unexplored. If preventing SARS-CoV-2 infections is crucial, the goal of averting cardiovascular involvement is not secondary. To this aim, it is important to have a low threshold of suspicion in the presence of clinical syndromes of recent onset, and immediately proceed to the precise and exhaustive characterization of the myocardial substrate by taking advantage of the tools available today. Recent study data show heart consequences such as myocarditis after recovery in patients who had no cardiac symptoms during their illness. In fact, in a study using cardiac magnetic resonance, it was observed that out of one hundred patients recovered from COVID-19 (treated at home or in the hospital), residual cardiac inflammation remained in 53% of patients. Another study considered 26 patients without comorbidity (professional athletes), of which 12 had mild symptoms, while 14 were asymptomatic. It was noted that four patients detected myocarditis without clinical symptoms, and two with mild symptoms, such as shortness of breath. Finally, none of the patients had an electrocardiogram, a blood test (troponin levels), or anything else that can show heart pain or other symptoms. It remains to be clarified when the inflammation of the myocardium develops after the exposure to coronavirus and when it remains active. This condition could compromise returning to normal physical activity after COVID-19 for a few months. According to the CDC (Centers for Disease Control and Prevention, USA), although most people with COVID-19 get better within weeks of the illness, some people experience post-COVID conditions (see Table 3) [119,120,121,122,123].

Thus, in the management of a patient with myocarditis, the follow-up of the clinical-instrumental picture and the reassessment of the ongoing evolution in the short and medium term are fundamental both for an accurate prognostic stratification in the long term and for the early identification of cases with a more negative natural history. Indeed, research is currently focusing on the long-term consequences after the viral infection is resolved.

### 4.8. Limitations

An authentic estimate of myocarditis in patients with COVID-19 infection is limited by several factors. First, given the profound impact of the COVID-19 pandemic on routine clinical practice and diagnostic approaches, several patients did not undergo further diagnostic investigations by means of cardiac magnetic resonance and/or endomyocardial biopsy. Second, it is important to emphasize that echographic findings are often unspecific, and abnormalities in wall motility as well as impaired ventricular function may be observed in cases of stress cardiomyopathy not necessarily dependent on viral infection. In addition, publication bias by the authors reporting some cases should also be taken into consideration. Finally, the patient number turns out to be a limitation. This is because the results in this review are based on conclusions from selected literature, which are thus lacking statistical significance because they are a simple cumulation of observations made in different contexts. However, and despite these limitations, data illustrated in this review should provide useful information for detecting trends and help making new assumptions on the potential onset of myocarditis in subjects with SARS-CoV-2 infection.

## 5. Conclusions

Our knowledge on management of COVID-19 infection is growing exponentially. Myocardial damage and myocarditis have been shown to be associated with increased morbidity and mortality, but there are still many unanswered questions and several areas to explore. Currently, most of the ongoing research focuses on respiratory complications, and little is known about the aspects characterizing COVID-19 myocarditis. Cases referred to previously considered healthy patients who have experienced heart failure linked to myocarditis, associated with SARS-CoV2 positivity, are growing. Nevertheless, data are, at present, still insufficient to explain how the new coronavirus should damage the heart muscle, how frequent is the heart inflammation associated with COVID-19 infection, and whether the available therapeutic strategy might be applied to all patients. In addition, several difficulties might be encountered in the differential diagnosis of acute myocarditis in the context of COVID-19 with respect to the SARS-CoV2-dependent myocarditis correlation. These uncertainties may explain the current lack of consensus on diagnostic and treatment algorithms in patients with a suspected COVID-19-induced acute myocarditis. By recapitulating the characteristics of the main studies that have analyzed COVID-19 patients with myocarditis, this review highlights the need for further research to improve our understanding of this pathological process and help define the most appropriate approach in these patients.

## Figures and Tables

**Table 1:** Characteristics of published cases of SARS-CoV-2-induced myocarditis.

Authors	Study Design	Sample of Study	Presenting Symptoms	Cardiac Biomarker	Results	Treatment
Asif and Ali [17]	Case report	64-year-old man	Dyspnea	Troponin: 0.17 ng/mL NT-proBNP: -	ECG: T Echo: - CMR: NR EMB/PM:NR	Aspirin, clopidogrel, heparin, azithromycin, hydroxychloroquine, meropenem, propofol, fentanyl, atracurium, vasopressin, tocilizumab
Asif and Ali [17]	Case report	71-year-old man	Fever, cough, and dyspnea	Troponin: 1.6 ng/mL NT-proBNP: -	ECG: ST, Q Echo: - CMR: NR EMB/PM:NR	Aspirin, clopidogrel, heparin, azithromycin, cefepime, vancomycin, tocilizumab, fentanyl, midazolam
Beri et al. [18]	Case report	72-year old man	Chest tightness, shortness of breath, cough, fatigue, and intermittent diarrhea	Troponin: - NT-proBNP: -	ECG: ST, TV Echo: ventricular dysfunction, cardiomegaly CMR: NR EMB/PM: NR	NR
Bernal-Torres et al. [19]	Case report	38-year-old woman	Palpitations, general malaise	Troponin: 1190 ng/mL NT-proBNP: 13,000 pg/mL	ECG: ST Echo: LVEF 30%, hypokinesia CMR: Edema+ Injury+ EMB/PM: NR	Methylprednisolone, immunoglobulin, hydroxychloroquine, azithromycin, lopinavir/ritonavir
Beşler and Arslan [20]	Case report	20-year-old man	Febrile sensation and chest pain	Troponin: 0.572 ng/mL NT-proBNP: 127 pg/L	ECG: - Echo: - CMR: Edema+ Injury+ EMB/PM:NR	Hydroxychloroquine, azithromycin, ceftriaxone, tigecycline, favipiravir, colchicine
Cizgici et al. [21]	Case report	78-year-old man	Chest pain and shortness of breath.	Troponin: 998.1 ng/mL NT-proBNP: 127 pg/mL	ECG: FA, ST Echo: - CMR: Edema+ Injury+ EMB/PM:NR	Furosemide, ACE-I, beta-blocker, COVID-19-specific therapy
Coyle et al. [22]	Case report	57-year-old man	Shortness of breath, fevers, cough, myalgia, decreased appetite, nausea, and diarrhea	Troponin: 7.33 ng/mL NT-proBNP: 1300 pg/mL	ECG: - Echo: Hypokinesia, LVEF 35–40% CMR: Edema+ Injury+ EMB/PM:NR	Hydroxychloroquine, methylprednisolone, azithromycin, ceftriaxone, colchicine
Craver et al. [23]	Case report	17-year-old man	Full cardiac arrest after a 2-day history of headache, dizziness, nausea, and vomiting	NR	ECG: NR Echo: NR CMR: NR EMB/PM: Immune cell infiltrate, necrosis	NR
Dolhnikoff et al. [24]	Case report	11-year-old boy	Fever, odynophagia, myalgia, and abdominal pain	Troponin: 0.342 ng/mL NT-proBNP: -	ECG: TV Echo: LVEF 31%, hypokinesia CMR: Edema+ Injury+ EMB/PM: viral particles in different cell lineages of the heart, including cardiomyocytes, endothelial cells, mesenchymal cells	Epinephrine, furosemide, ceftriaxone, azithromycin
Doyen et al. [25]	Case report	69-year-old man	Cough, fever, dyspnea	Troponin: 9002 ng/mL NT-proBNP: 22.600 pg/mL	ECG: T Echo: LVH CMR: Edema+ Injury+ EMB/PM: NR	Hydrocortisone, aspirin, fondaparinux
Fried et al. [26]	Case report	64-year-old woman	Chest pressure	Troponin: 18,6 ng/mL NT-proBNP: -	ECG: Sinusal tachy, ST Echo: LVEF 30%, pericardial effusion CMR: NR EMB/PM: NR	Hydroxychloroquine, dobutamine, IABP
Hu et al. [27]	Case report	37-year-old man	Chest pain, dyspnea, and diarrhea	Troponin: 10,000 ng/mL NT-proBNP: 2102 pg/mL	ECG: ST Echo: LVD, LVEF 27% CMR: NR EMB/PM:NR	Methylprednisolone, immunoglobulin, norepinephrine, diuretic, milrinone, pantoprazole, piperacillin/sulbactam
Inciardi et al. [28]	Case report	53-year-old woman	Fever and cough	Troponin: 0,24 ng/mL NT-proBNP: 5647 pg/mL	ECG: ST, T Echo: Hypokinesia, LVEF 40%, pericardial effusion CMR: Edema+ Injury+ EMB/PM: NR	Hydroxychloroquine, lopinavir/ritonavir, methylprednisolone, bisoprolol, kanrenone, furosemide
Irabien- Ortiz et al. [29]	Case report	59-year-old woman	Anginal chest pain in the absence of respiratory symptoms	Troponin: 1100 ng/mL NT-proBNP: 4421 pg/mL	ECG: ST Echo: LVH CMR: Edema+ Injury+ EMB/PM:NR	Immunoglobulins, methylprednisolone, ritonavir/lopinavir, interferon-B
Jacobs et al. [30]	Case report	48-year-old man	Fever, diarrhoea, cough, dysosmia, and dyspnea	Troponin: 14 932 ng/mL NT-proBNP: 9223 pg/mL	ECG: T Echo: LVH, hyperdynamic ventricular function CMR: NR EMB/PM: Immune cell infiltrate, edema, necrosis	Hydroxychloroquine, azithromycin, ECMO
Juusela et al. [31]	Case report	45-year-old woman, pregnant (39 weeks)	Contractions and emesis	Troponin: 0.046 ng/mL NT-proBNP: 114 pg/mL	ECG: - Echo: LVEF 40% CMR: NR EMB/PM:NR	Methylprednisolone, hydroxychloroquine, tocilizumab, cesarean
Juusela et al. [31]	Case report	26-year-old woman, pregnant (33 weeks)	Shortness of breath, dyspnea,	Troponin: 0.046 ng/mL NT-proBNP: <10 pg/mL	ECG: VT Echo: Hypokinesia, LVEF 40% CMR: - EMB/PM:NR	Metoprolol, cesarean
Kim et al. [32]	Case report	21-year-old woman	Coughing, sputum, diarrhea, and shortness of breath	Troponin: 1.26 ng/mL NT-proBNP: 1929 pg/mL	ECG: VE Echo: Severe LV systolic dysfunction CMR: Edema+ Injury+ EMB/PM: NR	NR
Luetkens et al. [33]	Case report	79-year-old man	Fatigue, shortness of breath, recurrent syncopes	Troponin: 63,5 ng/mL NT-proBNP: 1178 pg/mL	ECG: - Echo: Hypokinesia, LVEF 49% CMR: Edema+ Injury+ EMB/PM: NR	NR
Naneishvili et al. [34]	Case report	44-year-old woman	Fever, lethargy, muscle aches and two episodes of syncope	Troponin: 639 ng/mL NT-proBNP: -	ECG: FA Echo: LVEF 37%, LVH, pericardial effusion CMR: NR EMB/PM: NR	Methylprednisolone, remdesvir, amiodarone, milrinone, inotropic support
Oberweis et al. [35]	Case report	8-year-old male	Fever, coughing, weight loss, and severe fatigue	Troponin: 0.044 ng/mL NT-proBNP: 5112 pg/mL	ECG: ST Echo: LVEF 21%, pericardial effusion CMR: Edema+ Injury+ EMB/PM: NR	Heparin, dobutamine, immunoglobulins, milrinone, tocilizumab
Oleszak et al. [36]	Case report	52-year-old man	Cough, fever, shortness of breath, and a single episode of trace hemoptysis	Troponin: 0.017 ng/mL NT-proBNP: 1220 pg/mL	ECG: Sinusal tachy Echo: LVEF 25%, LVH CMR: NR EMB/PM: NR	Ceftriaxone, azithromycin, hydroxychloroquine, furosemide
Paul et al. [37]	Case report	35-year-old man	Chest pain and fatigue	Troponin: 2885 ng/mL NT-proBNP: -	ECG: repolarization changes Echo: - CMR: Edema+ Injury+ EMB/PM:NR	Ramipril, bisoprolol
Pavon et al. [38]	Case report	64-year-old man	Chest pain and dyspnea	Troponin: 1843 ng/L NT-proBNP: -	ECG: - Echo: LVEF 40% CMR: Edema+ Injury+ EMB/PM: NR	Piperacillin/tazobactam
Radbel et al. [39]	Case report	40-year-old man	Fever, dry cough, and dyspnea	Troponin: 30,39 ng/mL NT-proBNP: -	ECG: ST Echo: hypokinesia CMR: NR EMB/PM: NR	Hydroxychloroquine, azithromycin, tocilizumab
Rauf et al. [40]	Case report	5-year-old boy	Fever, abdominal pain and loose stools, bulbar conjunctivitis, and extremity edema	Troponin: 29 ng/mL NT-proBNP: 8000 pg/mL	ECG: - Echo: LVEF 35%, hypokinesia CMR: NR EMB/PM: NR	Inotropic support, immunoglobulins, aspirin, steroids, diuretics
Sala et al. [41]	Case report	43-year-old woman	Chest pain and dyspnea	Troponin: 106 ng/mL NT-proBNP: 512 pg/mL	ECG: ST Echo: Hypokinesia, LVEF 43% CMR: Edema+ Injury+ EMB/PM: Immune cell infiltrate, edema, necrosis	Lopinavir/ritonavir, hydroxychloroquine
Sardari et al. [42]	Case report	31-year-old man	Dyspnea on exertion and low-grade fever	Troponin: <0.03 ng/mL NT-proBNP: -	ECG: - Echo: Mild LV systolic dysfunction CMR: Edema+ Injury+ EMB/PM:NR	Bisoprolol, lisinopril
Tavazzi et al. [43]	Case report	69-year-old woman	Dyspnea, cough, and weakness	Troponin: 4332 ng/mL NT-proBNP: -	ECG: - Echo: LVD, LVEF 27% CMR: NR EMB/PM: viral particles within the interstitial cells of the myocardium, edema, necrosis	IABP, ECMO
Trogen et al. [44]	Case report	17-year-old man	Fever and neck pain, diarrhea	Troponin: 2.97 ng/mL NT-proBNP: 2124 pg/mL	ECG: T Echo: LVEF 40% CMR: Edema+ Injury+ EMB/PM: NR	Hydroxychloroquine, piperacillin-tazobactam, enoxaparin
Warchoł et al. [45]	Case report	74-year-old man	Palpitations, general malaise	Troponin: 102 ng/mL NT-proBNP: 2451 pg/mL	ECG: VT Echo: LVEF 25% CMR: Edema - Injury+ EMB/PM: NR	Metoprolol, amiodarone
Yuan et al. [46]	Case report	33-year-old man	Chest pain, fever, and muscle ache	Troponin: - NT-proBNP: -	ECG: - Echo: VT CMR: Edema+ Injury- EMB/PM:NR	NR
Zeng et al. [47]	Case report	63-year-old man	Shortness of breath and chest tightness after activity	Troponin: 11.37 g/L NT-proBNP: 22.600 pg/mL	ECG: Sinusal tachy Echo: dyskinesia, LVD, LVEF 32% CMR: NR EMB/PM: NR	High-flow oxygen, CCRTlopinavir-ritonavir, interferon α-1b, methylprednisolone, immunoglobulin, piperacillin-tazobactam
Cairns et al. [48]	Case report	58-year-old female	Developed cardiogenic shock after fever, diarrhoea, and vomiting	Troponin 3532.9 ng/L NT-proBNP: NR	ECG: NR Echo: TTE showed 3–4 cm pericardial effusion with evidence of cardiac tamponade CMR: NR EMB/PM: NR	Pericardial drain, vasopressor support, intravenous amoxicillin, oral doxycycline initially, escalated to piperacillin/tazobactam, furosemide, intravenous immunoglobulin (2 g/kg)
Beaudry et al. [49]		15-year-old female	Epigastric pain, nasal congestion, and rhinorrhea. Pyrexia (38.9°C), tachycardia, and worsening respiratory failure	Troponin 2.48 ng/mL NT-proBNP: 8328 pg/mL	ECG: sinus tachycardia Echo: severely diminished LV ejection CMR: NR EMB/PM: cardiomyocytes with interstitial lymphoplasmacytic infiltrate and mixed inflammation surrounding cardiac vessels	Mechanical ventilation (intensive care unit), norepinephrine infusion cefepime, metronidazole, doxycycline
Garot J et al. [50]	69-year-old man	Fever, asthenia, and abdominal pain, hypotension, hypoxemia, anuria, and multi-organ failure (MOF)	Troponin 8066 pg/mL NT-proBNP: NR		ECG: no signs of ischemia Echo: non-dilated and severe, diffuse LV hypokinesia (LV ejection fraction: 30%) CMR: NR EMB/PM: Abundant myocardial edema, interstitial inflammation (predominance of mononucleated leucocytes) associated with cardiomyocytes dystrophies and strong granular cytoplasmic staining of cardiomyocytes for SARS coronavirus nucleocapsid protein	Veno-arterial unit and extracorporeal membrane oxygenation (VA-ECMO), noradrenalin, dobutamine

Abbreviations: CMR—cardiac magnetic resonance imaging; ECG—electrocardiogram; Echo—echocardiogram; LVH—left ventricular hypertrophy; LVD—left ventricular dilated; NR—not reported; NT-pro-BNP—N-terminal pro B-type natriuretic peptide; EMB—endomyocardial biopsy; PM—post-mortem; ST—ST-segment changes; T—T-wave changes; VT—ventricular tachycardia; CRRT—continuous renal replacement therapy; “+”—abnormal or elevated; “-”—normal.

**Table 2:** The connection between myocardial injury and COVID-19 as discussed in the relevant studies.

Authors	Study Design	Sample of Study	Results	Treatment	Conclusion
Buja et al. [51]	Case series	23	Lymphocytic myocarditis, lymphocytic pericarditis, multifocal acute injury of cardiomyocytes without inflammatory cellular infiltrates	N/A	COVID-19 is a systemic disease with major involvement of the lungs and heart.
Chen et al. [52]	Retrospective study	99	Pneumonia, abnormal CT, cardiac injury, secondary infection	Mechanical respirators and circulatory support systems, including IABP, Impella, and ECMO	COVID-19 infection might deteriorate rapidly, eventually followed by fulminant myocarditis. More attention should be paid to patients with extremely increased cardiac troponin I levels and new-onset arrhythmias.
Deng et al. [53]	Retrospective study	112	High troponin level and pulmonary hypertension indicating myocardial injury	N/A	Myocardial injury is due to systemic consequences rather than direct injury by Sars-CoV2.
Escher et al. [54]	Retrospective study	104	EMB: active myocarditis according to the Dallas criteria, necrosis	N/A	Evidence of SARS-CoV-2 genomes detection in EMB.
Guo et al. [55]	Retrospective single-center case	187	Hypertension, coronary heart disease, cardiomyopathy, elevated TnT level exhibited myocardial injury	Antiviral therapy, antibiotics, respiratory support	Myocardial injury is essentially connected with the lethal outcome.
Halushka et al. [56]	Meta-analysis	22 studies with 277 patients	Macro or microvascular thrombi, inflammation, or intraluminal megakaryocytes	N/A	COVID-19-related cardiac histopathological findings are common, while myocarditis is rare.
Han et al. [57]	Single-center study	273	High blood levels of CKMB, MYO, Ultra-TnI, NTproBNP indicating acute cardiac injury	Monitoring of the myocardiac enzyme profiles	Higher concentrations of CK-MB, MYO, ultra-TnI, and NT- proBNPin venous blood signified the severity and case fatality rate (CFR) of COVID-19.
Khalid et al. [58]	Case series	2	Abnormal ECG and Echo, high blood levels of TnI, and NTproBNP	Dual antiplatelet therapy, heparin, dobutamine, norepinephrine, ECMO	Cases of COVID-19 infection with myocardial involvement may have distinct mechanistic pathways and outcomes.
Nunes Duarte-Neto et al. [59]	Case series	10	Hypertrophy of cardiomyocytes, myocardial fibrosis, interstitial edema fibrin thrombi (2 mild lymphomononuclear myocarditis)	N/A	COVID-19 is a systemic disease with major effects in the lungs and the involvement of various organs including the heart.
Puntmann et al. [60]	Cohort study	100	hsTnT values significantly elevated, EMB: active lymphocytic inflammation, CMR: myocardial fibrosis and/or edema	Antiviral, antibiotic, steroid, hydrochloroquine	The combination of histological findings as well as the increase relative to age-matched, sex-matched, and risk-factor-matched controls makes this a COVID-19-related inflammatory process.
Rajpal et al. [61]	Prospective study	26	CMR: myocardial edema and myocardial injury, pericardial effusion	N/A	CMR may provide an excellent risk-stratification assessment for myocarditis in athletes who have recovered from COVID-19.
Ruan et al. [62]	Retrospective multi-center study	150	Predictors of a fatal outcome included age, underlying diseases, secondary infection, and elevated inflammatory indicators in the blood	N/A	The infection of SARS-CoV-2 may cause fulminant myocarditis, and patients with cardiovascular diseases have a significantly increased risk of death when they are infected with SARS-CoV-2.
Shi et al. [63]	Cohort study	416	Abnormal ECG, bilateral pneumonia, ground-glass opacity, mottling	Antiviral therapy, oxygen, glucocorticoids, intravenous immunoglobulin therapy, antibiotic therapy	The data and findings support cardiac injury as a complication in COVID-19 patients.
Yang et al. [64]	Meta-analysis	6 studies with 1527 patients	Hypertension, cardiac cerebrovascular disease, and diabetes	N/A	COVID-19 might aggravate the damage to the heart.

Abbreviations: CMR—cardiac magnetic resonance imaging; ECG—electrocardiogram; Echo—echocardiogram; LVH—left ventricular hypertrophy; LVD—left ventricular dilated; NR—not reported; NT-pro-BNP—N-terminal pro B-type natriuretic peptide; EMB—endomyocardial biopsy; PM—post-mortem; ST—ST-segment changes; T—T-wave changes; VT—ventricular tachycardia; CRRT—continuous renal replacement therapy; “+”—abnormal or elevated; “-”—normal.

**Table 3:** Health Issues (post-COVID 19 conditions) for infected individuals according to CDC (modified from source: https://www.cdc.gov/coronavirus/2019-ncov/long-term-effects.html (accessed on 20 May 2021).

Types of Post-COVID Conditions		
Long COVID-19	COVID-19 Multiorgan Effects	COVID-19 Treatment or Hospitalization Effects
A range of symptoms that can last weeks or months after first being infected or can appear weeks after infection. Can happen to anyone who has had COVID-19, even if the illness was mild, or they had no symptoms. Tiredness or fatigue, difficulty thinking or concentrating (sometimes referred to as “brain fog”), headache, loss of smell or taste, orthostatic dizziness, fast-beating or pounding heart (also known as heart palpitations), chest pain, difficulty breathing or shortness of breath, cough, joint or muscle pain, depression or anxiety, fever, symptoms that get worse after physical or mental activities.	Multiorgan effects can affect most, if not all, body systems, including heart, lung, kidney, skin, and brain functions. Can also include conditions that occur after COVID-19, like multisystem inflammatory syndrome (MIS) and autoimmune condition.	Longer-term effects of COVID-19 treatment or hospitalization (some of these effects are similar to those related to hospitalization for other respiratory infections or other conditions). Can include severe weakness and post-traumatic stress disorder (PTSD involves long-term reactions to a stressful event), and post-intensive care syndrome (PICS).

## Data Availability

Not applicable.
